# Supplementary material for: Rectifying artificial nanochannels with multiple interconvertible permeability states
Source: Nat Commun. 2024 Mar 6;15:2051. doi: 10.1038/s41467-024-46312-w (PMC10918189; doi:10.1038/s41467-024-46312-w)
Supplement: Supplementary file 1 — Supplementary Information [file 41467_2024_46312_MOESM1_ESM.pdf]

Supplementary information for

Rectifying artificial nanochannels with multiple interconvertible permeability states

Ruocan Qian[*,1,2,3,4,+], Mansha Wu[1,2,3,4,+], Zhenglin Yang[5], Yuting Wu[5], Weijie Guo[6], Zerui Zhou[1,2,3,4], Xiaoyuan Wang[1,2,3,4], Dawei Li[1,2,3,4], and Yi Lu[*,5,6]

[1]Key Laboratory for Advanced Materials. East China University of Science and Technology, Shanghai 200237, P. R. China

[2]Feringa Nobel Prize Scientist Joint Research Center, Joint International Laboratory for Precision Chemistry. East China University of Science and Technology, Shanghai 200237, P. R. China

[3]Frontiers Science Center for Materiobiology & Dynamic Chemistry. East China University of Science and Technology, Shanghai 200237, P. R. China

[4]School of Chemistry and Molecular Engineering, East China University of Science and Technology, Shanghai 200237, P. R. China

[5]Department of Chemistry, University of Texas at Austin, Austin, Texas 78712, USA

[6]Department of Molecular Biosciences, University of Texas at Austin, Austin, Texas 78712, USA

[+]These authors contribute equally to this work.

[*]Corresponding author:

Ruocan Qian (ruocanqian@ecust.edu.cn)

Yi Lu (yi.lu@utexas.edu)

Figures:

Supplementary Fig. 1 | Schematic illustration showing the synthesis process of nanopipettes functionalized with DNAzyme via aldehyde modification to bind with amino group-labeled DNAzyme.

Supplementary Fig. 2 | Schematic illustration showing the synthesis process of nanopipettes functionalized with DNAzyme via Au cladding modification to bind with thiol-labeled DNAzyme.

Supplementary Fig. 3 | UV-vis characterization of $HAuCl_4$ before and after Au cladding modification.

Supplementary Fig. 4 | FL spectra of FAM-labeled SH-DNAzyme before and after ligation.

Supplementary Fig. 5 | Current-voltage (*I*-*V*) curves measured in nanopipettes modified by DNAzyme with different lengths.

Supplementary Fig. 6 | Rectification ratio measured in nanopipettes modified by DNAzyme with different concentrations.

Supplementary Fig. 7 | Current-voltage (*I*-*V*) curves measured in nanopipettes modified by DNAzyme chain-4 and chain-5 with different lengths.

Supplementary Fig. 8 | Wettability characterization of DNA modified surfaces at state i and state ii.

Supplementary Fig. 9 | Mg^{2+}-specific DNAzyme mediated specific substrate cleavage evaluated by agarose gel electrophoresis.

Supplementary Fig. 10 | Availability and specificity of the DNAzyme-based switching between hydrophobic and hydrophilic evaluated by inactive DNAzyme 1 and different metal ions.

Supplementary Fig. 11 | Photos showing the electrochemical monitoring system.

Supplementary Fig. 12 | Wettability characterization of DNA modified surfaces at state iii and state iv.

Supplementary Fig. 13 | Zn^{2+}-specific DNAzyme mediated specific substrate cleavage evaluated by agarose gel electrophoresis.

Supplementary Fig. 14 | Availability and specificity of the DNAzyme-based switching between negative and positive rectification evaluated by inactive DNAzyme 2 and different metal ions.

Supplementary Fig. 15 | UV-vis, FL characterizations and confocal microscopic images of MB dyes.

Supplementary Fig. 16 | Nyquist plots and contact angles of state 1, 2, 3 and 4.

Supplementary Fig. 17 | Metal ion selectivity of DNAzyme evaluated by agarose gel electrophoresis.

Supplementary Fig. 18 | Mg^{2+} / Zn^{2+}-specific DNAzyme mediated specific substrate cleavage evaluated by agarose gel electrophoresis (the reversible transfer between four different states).

Supplementary Fig. 19 | Microscopic cell images showing the outflow of FITC dye molecules into single living HeLa cells.

Supplementary Fig. 20 | Microscopic cell images showing the outflow of MB dye molecules into single living HeLa cells.

Supplementary Fig. 21 | Microscopic cell images showing the outflow of TB dye molecules into single living HeLa cells.

Supplementary Fig. 22 | Microscopic cell images showing the outflow of FITC dye

molecules into single living HeLa cells.
Supplementary Fig. 23 | Microscopic cell images showing the outflow of MB dye molecules into single living HeLa cells.
Supplementary Fig. 24 | Microscopic cell images showing the outflow of TB dye molecules into single living HeLa cells.
Supplementary Fig. 25 | The simulated geometry of the nanopipette.
Supplementary Fig. 26 | The simulated geometry of the nanopipette (not to scale).
Supplementary Fig. 27 | The distribution of potential and electric field in the simulated nanopipette.
Supplementary Fig. 28 | FL intensity comparison histogram of TB before and after the treatment of TB dye molecules.
Supplementary Fig. 29 | Microscopic cell images showing TB treated cells in different states.
Supplementary Fig. 30 | Specific recognition between miR-21 and hp-seed evaluated by agarose gel electrophoresis.
Supplementary Fig. 31 | FL intensity comparison histogram of FAM before and after miR-21 silencing.
Supplementary Fig. 32 | Microscopic cell images showing the proliferation of HeLa cells treated without or with miR-21 silencing.
Supplementary Fig. 33 | FL intensity comparison histogram of Fluo-4 before and after the treatment of Ca^{2+}.
Supplementary Fig. 34 | Microscopic cell images showing the FL of fluo-4 before and after the treatment of Zn^{2+} ions.
Supplementary Fig. 35 | Microscopic cell images showing the detection of Ca^{2+} based on Fluo-4 probe in PC-12 cells.
Supplementary Fig. 36 | Microscopic cell images showing the spontaneous FL of HeLa cells and PC-12 cells.

Tables:

Supplementary Table 1 | State conversion of artificial nanochannels.
Supplementary Table 2 | Oligonucleotides sequences.
Supplementary Table 3 | Parameters for the fabrication of nanopipettes using a P-2000 laser.
Supplementary Table 4 | The detailed simulation parameters of the nanopipette model.
Supplementary Table 5 | The detailed boundary conditions used in the nanopipette model.

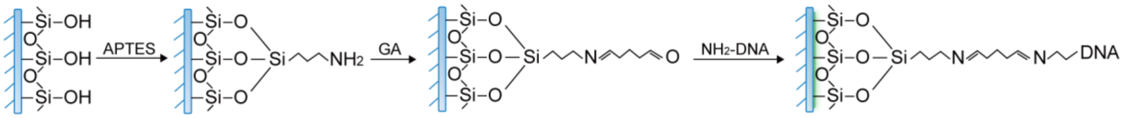

Supplementary Fig. 1 | Schematic illustration showing the synthesis process of nanopipettes functionalized with DNAzyme via aldehyde modification to bind with amino group-labeled DNAzyme. First, 5% aminopropyl triethoxysilane (APTES) solution was injected into the tip of nanopipettes for 30 min, and then the nanopipettes were washed with ethanol for three times before placed in a vacuum drying oven for another 30 min at 110 ℃. Next, 2.5% glutaric dialdehyde (GA) solution was injected into the tip of nanopipettes for 10 h, and then the nanopipettes were washed with water for three times. Finally, amino group-labeled DNAzyme solution (1 μM) was injected into the nanopipettes and incubated for 6 h to achieve DNAzyme modification.

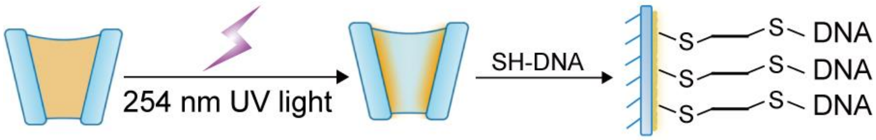

Supplementary Fig. 2 | Schematic illustration showing the synthesis process of nanopipettes functionalized with DNAzyme via Au cladding modification to bind with thiol-labeled DNAzyme. First, ethanol and chlorauric acid ($HauCl_4$, 8 mM) were mixed according to the volume ratio of 1:3 and then injected into the tip of nanopipettes for 2.5 h under 254 nm UV light irradiation. Then the nanopipettes were placed in a drying oven for 30 min at 100 ℃ to obtain the inner gold film after washing with ethanol and water. Next the nanopipettes were filled with the mixed solution of thiol-labeled DNAzyme (1 μM) and tris(2-carboxyethyl)-phosphine (TCEP, 1 mM) and incubated for 12 h to achieve DNAzyme modification.

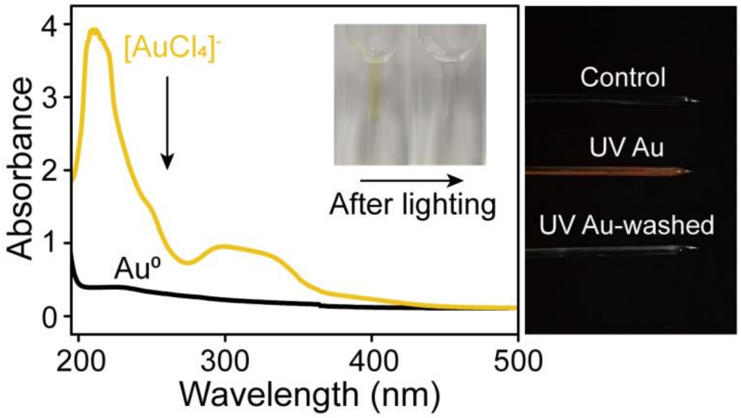

Supplementary Fig. 3 | UV-vis characterization of $HAuCl_4$ before and after Au cladding modification. Left: UV-vis spectra of $HAuCl_4$ before (yellow curve) and after (black curve) Au cladding modification. Inset: the photo showing the color change of $HAuCl_4$ solution before and after UV irradiation. Right: the photo showing three nanopipettes under different conditions. Control: bare nanopipette; UV Au: nanopipette functionlized with Au layer under UV irradiation; UV Au-washed: nanopipette functionlized with Au layer after washing with ethanol and water for two times. Source data are provided as a Source Data file.

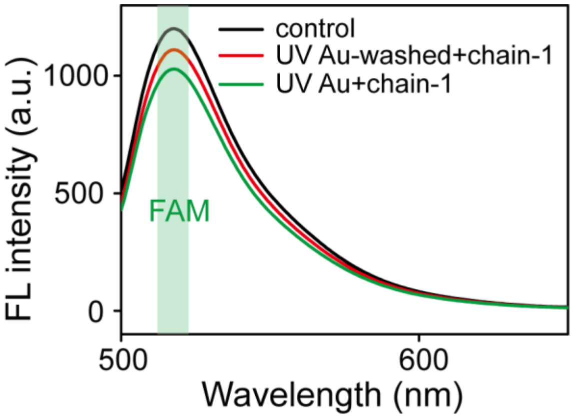

Supplementary Fig. 4 | FL spectra of FAM-labeled SH-DNAzyme before and after ligation. In order to prove the successful modification of DNAzyme on the inner surface of nanopipettes with Au cladding, chain-1 was injected into the nanopipettes for ligation, and then the DNAzyme solution before and after reaction was sent for FL spectra characterization. Control: 2 μM FAM-labeled SH-DNAzyme dissolved in PBS before reaction. UV Au-washed+chain-1: nanopipette functionlized with Au layer after washing with ethanol and water for two times was used for FAM-labeled SH-DNAzyme ligation. The DNAzyme solution after reaction was extracted and sent for FL spectroscopy. UV Au+chain-1: nanopipette functionlized with Au layer without washing was used for FAM-labeled SH-DNAzyme ligation. The DNAzyme solution after reaction was extracted and sent for FL spectroscopy. Source data are provided as a Source Data file.

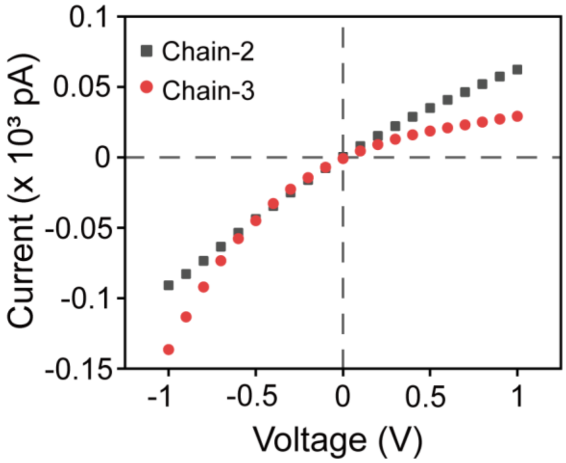

Supplementary Fig. 5 | Current-voltage (*I*-*V*) curves measured in nanopipettes modified by DNAzyme with different lengths. To improve the hybridization efficiency between DNAzyme and nanopipettes, chian-2 and chain-3 were used for inner surface modification, and the ion rectification curve (ICR) of the functionalized nanopipettes were measured separately. Source data are provided as a Source Data file.

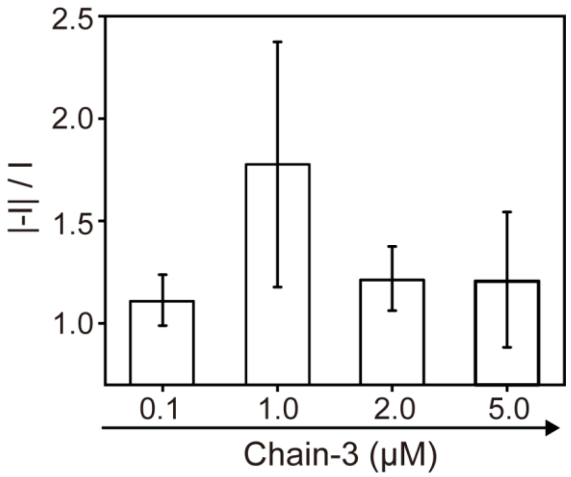

Supplementary Fig. 6 | Rectification ratio measured in nanopipettes modified by DNAzyme with different concentrations. After the optimization of modification methods and the length of DNAzyme, the concentration of DNAzyme was further optimized. Chain-3 with different concentrations (0.1 μM, 1.0 μM, 2.0 μM, 5.0 μM) were used for nanopipette modification respectively. The rectification ratios of the modified nanopipettes were tested separately. Error bars represent the standard deviation of three independent experimental repeats and the measure of the centre represents their corresponding mean value. Source data are provided as a Source Data file.

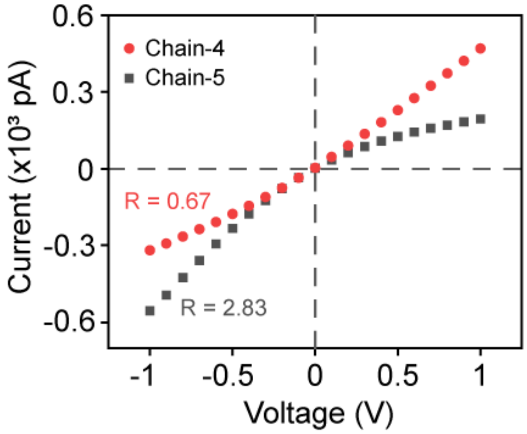

Supplementary Fig. 7 | Current-voltage (I-V) curves measured in nanopipettes modified by chain-4 and chain-5 with different lengths. The inherent phosphate backbone structure of DNAzyme makes it negatively charged in solution. Nevertheless, the introduction of spacer could enhance the hybridization density to increase the binding efficiency between DNAzyme and nanopipettes, leading to more explosure of amino groups, as the ICR ratio (the ratio of current at -1 V to current at +1 V) changed from 2.83 (negative) to 0.67 (positive). Source data are provided as a Source Data file.

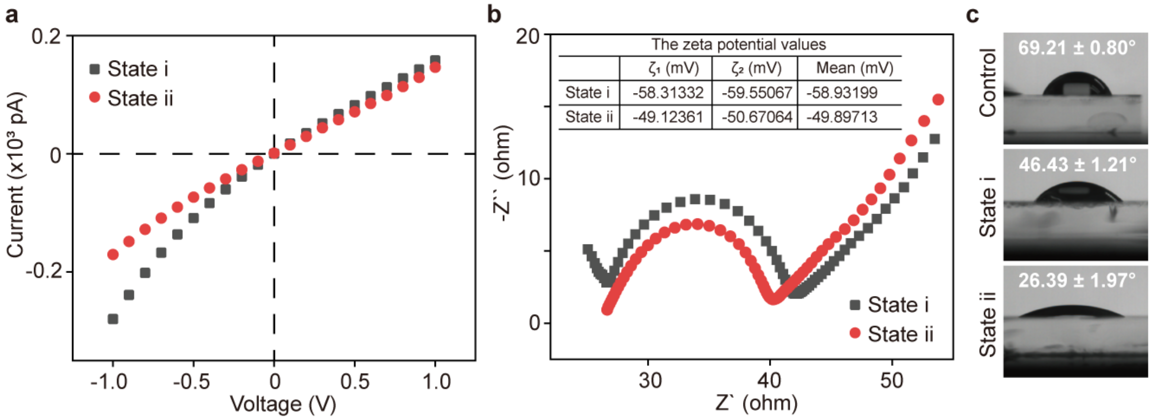

Supplementary Fig. 8 | Wettability characterization of DNA modified surfaces at state i and state ii. a, Current-voltage (*I-V*) curves measured in a wettability-reversal nanochannel before and after the treatment of Mg^{2+}. **b,** Nyquist plot of DNA modified surfaces (planar glass plates modified with DNAzyme 1/Sub-1) without and with the treatment Mg^{2+} using 10 mM $[Fe(CN)_6]^{3-}/[Fe(CN)_6]^{4-}$ as redox mediators. The inset table: the zeta potential values of DNA modified surfaces (planar glass plates modified with DNAzyme 1/Sub-1) without and with the treatment of Mg^{2+}. **c,** Photographs of the water droplets on DNA modified surfaces (planar glass plates modified with DNAzyme 1/Sub-1) without and with the treatment of Mg^{2+}. The uncropped water droplets are shown in Source Data. The average contact angle value was obtained from the value detected at three different locations (n=3 independent experiments and the data were presented as the mean values ± SDs). Source data are provided as a Source Data file.

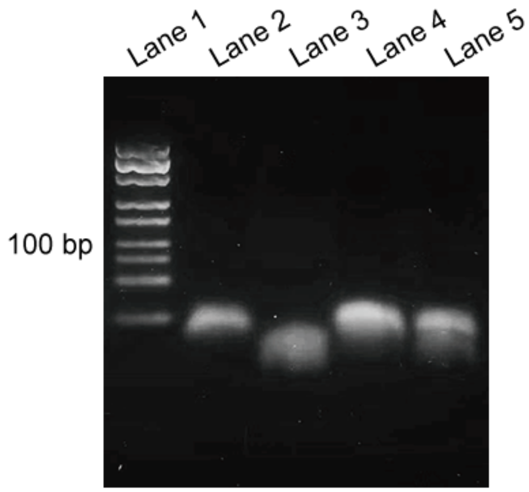

Supplementary Fig. 9 | Mg^{2+}-specific DNAzyme mediated specific substrate cleavage evaluated by agarose gel electrophoresis. From lanes 1 to 5: ladder (lane 1), DNAzyme 1 (lane 2), substrate 1 (lane 3), mixture of DNAzyme 1 / substrate 1 (lane 4), and mixture of DNAzyme 1 / substrate 1 treated with Mg^{2+} (lane 5). The agarose gel was run in 1x Tris-Borate-EDTA buffer at 100 V for 35 min and then stained by ethidium bromide. The gel was imaged on a Bio-Rad molecular imager under blue light. The uncropped gel is shown in Source Data. Source data are provided as a Source Data file.

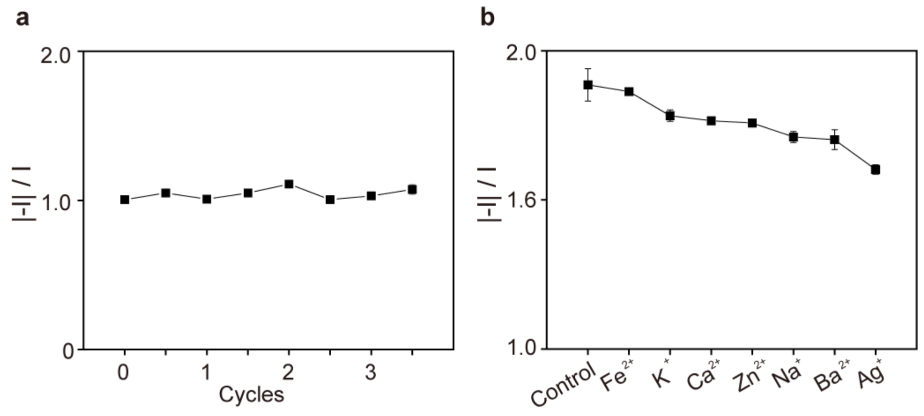

Supplementary Fig. 10 | Availability and specificity of the DNAzyme-based switching between hydrophobic and hydrophilic evaluated by inactive DNAzyme 1 and different metal ions. a, Rectification ration measured in nanopipettes modified by inactive DNAzyme 1 (chain-6) and sub-1 after 3 rounds of addition of Mg^{2+} and sub-1. **b,** Rectification ration measured in nanopipettes modified by DNAzyme 1 and sub-1 upon the addition of different metal ions (Fe^{2+}, K^{+}, Ca^{2+}, Zn^{2+}, Na^{+}, Ba^{2+}, Ag^{+} and all the metal ions were 2 μM). Error bars in a and b represent the standard deviation of three independent experimental repeats and the measure of the centre represents their corresponding mean value. Source data are provided as a Source Data file.

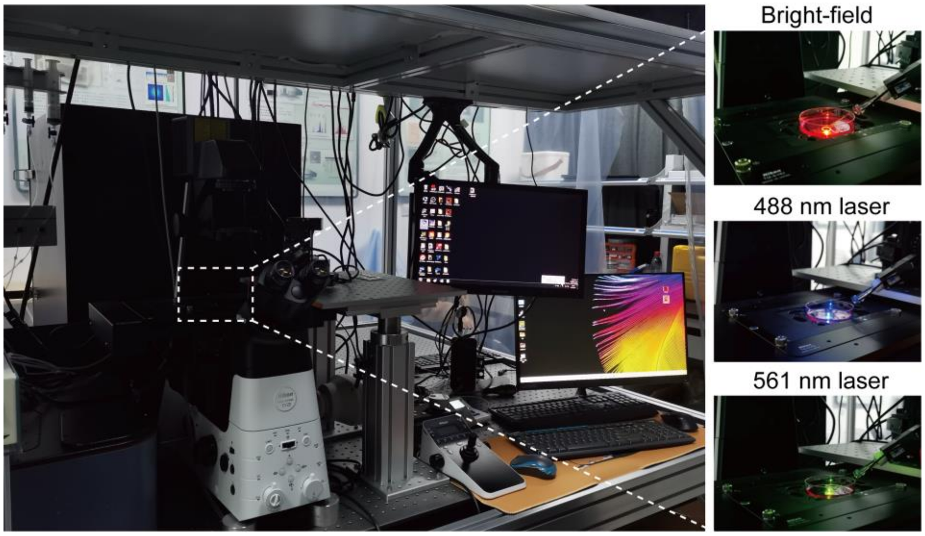

Supplementary Fig. 11 | Photos showing the electrochemical monitoring system. The nanopipette was fixed on a holder and connected to the headstage of the Axopatch 200B device. A previously designed protocol was edited to perform electrochemical experiments with pClamp 10.7 (Axon Instrument, Forest City, USA) run on a PC. The record mode was gap-free with a sampling frequency of 100 kHz and a 5 kHz low-pass Bessel filter. The nanopipette was controlled to move along the X, Y, and Z directions when the micromanipulation system was turned on, until the nanopipette tip was moved approaching the targeted location. The whole process was monitored under an inverted microscope.

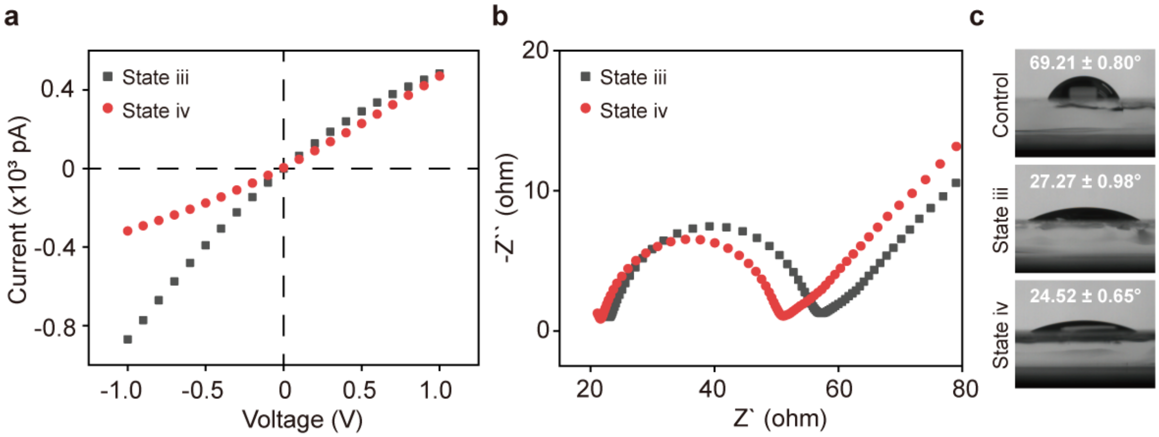

Supplementary Fig. 12 | Wettability characterization of DNA modified surfaces at state iii and state iv. a, Current-voltage (*I*-*V*) curves measured in a wettability-reversal nanochannel before and after the treatment of Zn^{2+}. **b,** Nyquist plot of DNA modified surfaces (planar glass plates modified with DNAzyme 2/Sub-2) without and with the treatment Zn^{2+} using 10 mM $[Fe(CN)_6]^{3-}$/$[Fe(CN)_6]^{4-}$ as redox mediators. **c,** Photographs of the water droplets on DNA modified surfaces (planar glass plates modified with DNAzyme 2/Sub-2) without and with the treatment of Zn^{2+}. The uncropped water droplets are shown in Source Data. The average contact angle value was obtained from the value detected at three different locations (n=3 independent experiments and the data were presented as the mean values ± SDs). Source data are provided as a Source Data file.

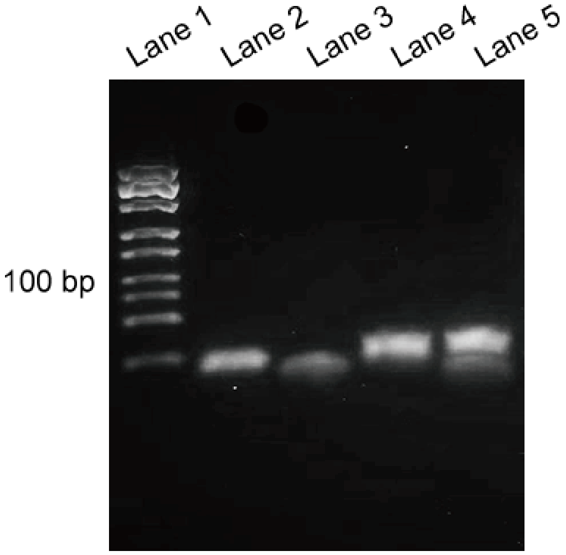

Supplementary Fig. 13 | Zn^{2+}-specific DNAzyme mediated specific substrate cleavage evaluated by agarose gel electrophoresis. From lanes 1 to 5: ladder (lane 1), DNAzyme 2 (lane 2), substrate 2 (lane 3), mixture of DNAzyme 2 / substrate 2 (lane 4), and mixture of DNAzyme 2 / substrate 2 treated with Zn^{2+} (lane 5). The agarose gel was run in 1x Tris-Borate-EDTA buffer at 100 V for 35 min and then stained by ethidium bromide. The gel was imaged on a Bio-Rad molecular imager under blue light. The uncropped gel is shown in Source Data. Source data are provided as a Source Data file.

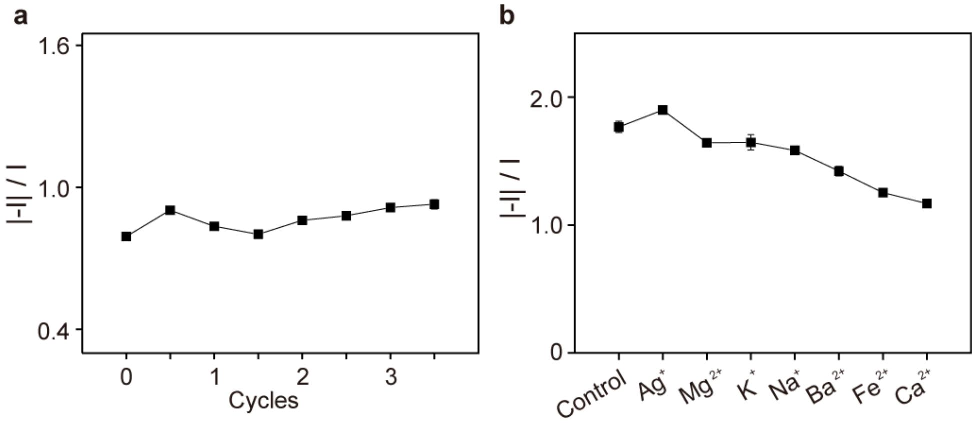

Supplementary Fig. 14 | Availability and specificity of the DNAzyme-based switching between negative and positive rectification evaluated by inactive DNAzyme 2 and different metal ions. a, Rectification ration measured in nanopipettes modified by inactive DNAzyme 2 (chain-7) and sub-2 after 3 rounds of addition of Zn^{2+} and sub-2. **b,** Rectification ration measured in nanopipettes modified by DNAzyme 2 and sub-2 upon the addition of different metal ions (Ag^{+}, Mg^{2+}, K^{+}, Na^{+}, Ba^{2+}, Fe^{2+}, Ca^{2+} and all the metal ions were 2 µM). Error bars in a and b represent the standard deviation of three independent experimental repeats and the measure of the centre represents their corresponding mean value. Source data are provided as a Source Data file.

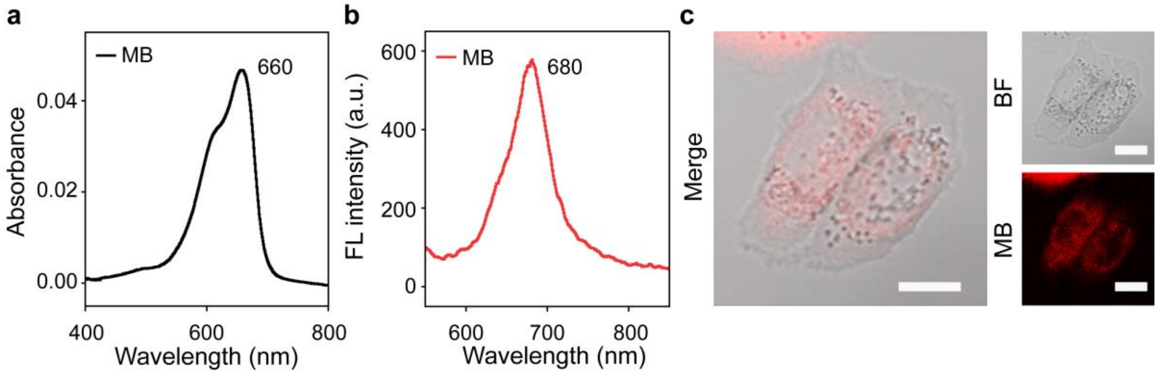

Supplementary Fig. 15 | UV-vis, FL characterizations and confocal microscopic images of MB dyes. a, UV-vis spectra of MB dyes. **b,** FL spectra of MB dyes. **c,** Microscopic cell images of living HeLa cells incubated with MB dyes. BF: bight-field. MB: red fluorescence. Merge: mixed red & bright-field channel. Scale bar: 20 μm. Source data are provided as a Source Data file.

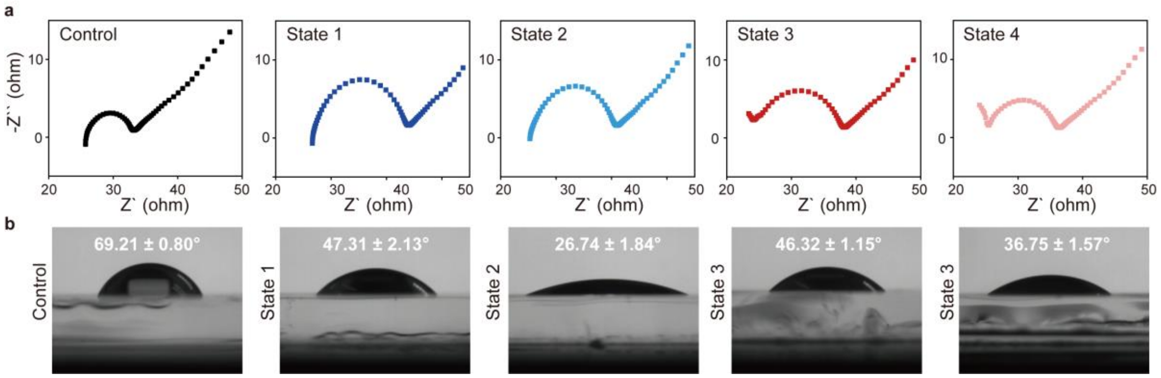

Supplementary Fig. 16 | Nyquist plots and contact angles of state 1, 2, 3 and 4. a, Nyquist plots of DNA modified surfaces of state 1, 2, 3 and 4 using 10 mM $[Fe(CN)_6]^{3-}$/$[Fe(CN)_6]^{4-}$ as redox mediators. **b,** Photographs of the water droplets on DNA modified surfaces of state 1, 2, 3 and 4. The uncropped water droplets are shown in Source Data. The average contact angle value was obtained from the value detected at three different locations (n=3 independent experiments and the data were presented as the mean values ± SDs). Source data are provided as a Source Data file.

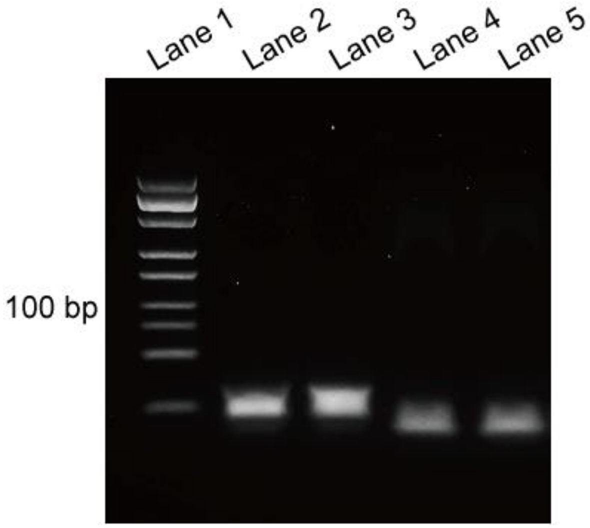

Supplementary Fig. 17 | Metal ion selectivity of DNAzyme evaluated by agarose gel electrophoresis. From lanes 1 to 5: ladder (lane 1), mixture of DNAzyme 2 / substrate 2 (lane 2), mixture of DNAzyme 2 / substrate 2 treated with Mg^{2+} (lane 3), mixture of DNAzyme 1 / substrate 1 (lane 4), and mixture of DNAzyme 1 / substrate 1 treated with Zn^{2+} (lane 5). The agarose gel was run in 1x Tris-Borate-EDTA buffer at 100 V for 35 min and then stained by ethidium bromide. The gel was imaged on a Bio-Rad molecular imager under blue light. The uncropped gel is shown in Source Data. Source data are provided as a Source Data file.

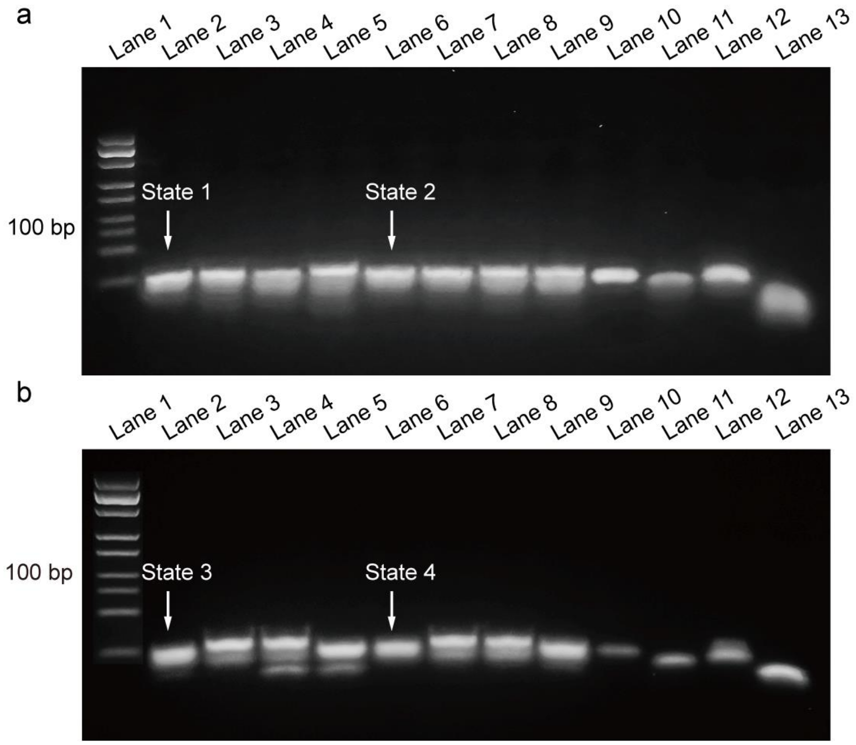

Supplementary Fig. 18 | Mg^{2+} / Zn^{2+}-specific DNAzyme mediated specific substrate cleavage evaluated by agarose gel electrophoresis (the reversible transfer between four different states). a, From lanes 1 to 13: ladder (lane 1); mixture of double chain of DNAzyme 1 / substrate 1 and DNAzyme 2 / substrate 2 (state 1, lane 2), state 1 treated with Mg^{2+} (state 2, lane 3), state 1 treated with Zn^{2+} (state 3, lane 4), state 1 treated with Zn^{2+} and Mg^{2+} (state 4, lane 5); mixture of DNAzyme 1 and double chain of DNAzyme 2 / substrate 2 (state 2, lane 6), state 2 treated with substrate 1 (state 1, lane 7), state 2 treated with substrate 1 and Zn^{2+} (state 3, lane 8), state 2 treated with Zn^{2+} (state 4, lane 9); DNAzyme 2 (lane 10), substrate 2 (lane 11), DNAzyme 1 (lane 12), substrate 1 (lane 13). **b,** From lanes 1 to 13: ladder (lane 1); mixture of double chain of DNAzyme 1 / substrate 1 and DNAzyme 2 (state 3, lane 2), state 3 treated with substrate 2 (state 1, lane 3), state 3 treated with substrate 2 and Mg^{2+} (state 2, lane 4), state 3 treated with Mg^{2+} (state 4, lane 5); mixture of DNAzyme 1 and DNAzyme 2 (state 4, lane 6), state 4 treated with substrate 1 and substrate 2 (state 1, lane 7), state 4 treated with substrate 2 (state 3, lane 8), state 4 treated with substrate 1 (state 2, lane 9); DNAzyme 2 (lane 10), substrate 2 (lane 11), DNAzyme 1 (lane 12), substrate 1 (lane 13). The agarose gel was run in 1x Tris-Borate-EDTA buffer at 100 V for 35 min and then stained by ethidium bromide. The gel was imaged on a Bio-Rad molecular imager under blue light. The uncropped gels are shown in Source Data. Source data are provided as a Source Data file.

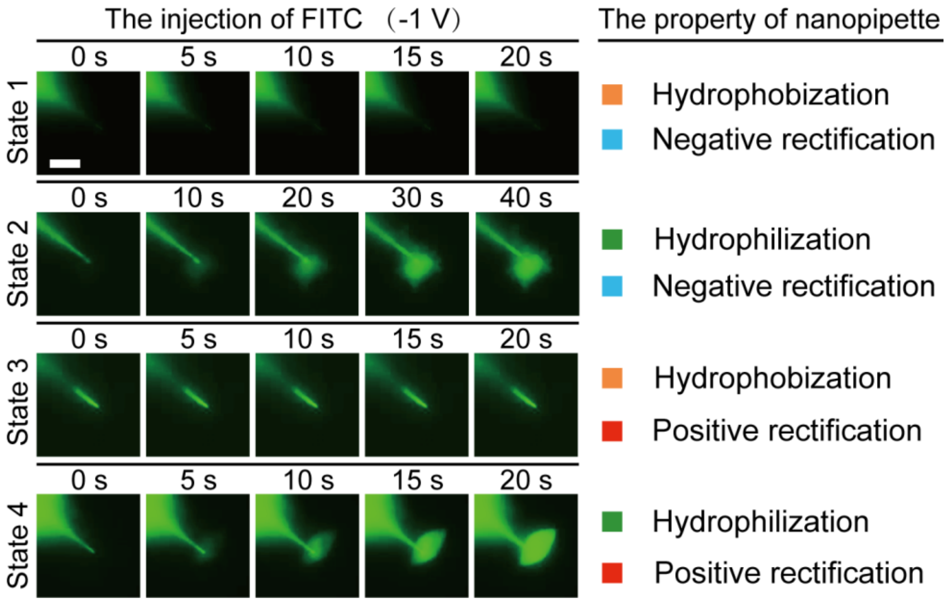

Supplementary Fig. 19 | Microscopic cell images showing the outflow of FITC dye molecules into single living HeLa cells. FITC solution (5 mg/mL) was injected into the tip of nanopipettes with different states at -1 V. State 1 and state 3 with cholesterol tail on the surface of nanochannel were hydrophobic, showing greater attraction to FITC. Upon the treatment of 1 μM Mg^{2+} (30 min) to induce substrate 1 cleavage, the state of the nanochannel changed from hydrophobic to hydrophilic (state 2 and 4), showing decreased attraction to FITC. Scale bar: 20 μm. State 1: hydrophobization, negative rectification. State 2: hydrophilization, negative rectification. State 3: hydrophobization, positive rectification. State 4: hydrophilization, positive rectification. Scale bar: 20 μm.

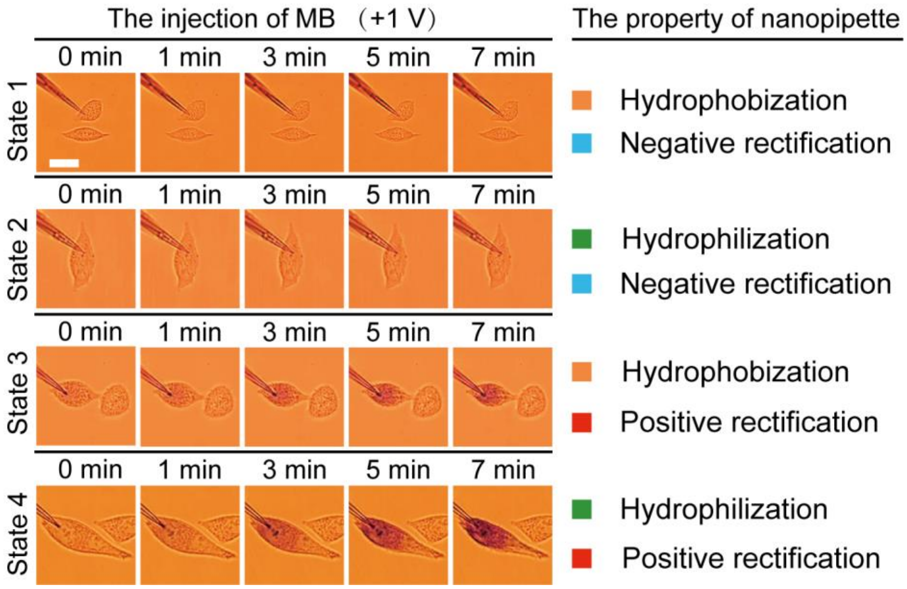

Supplementary Fig. 20 | Microscopic cell images showing the outflow of MB dye molecules into single living HeLa cells. MB solution (5 mg/mL) was injected into the tip of nanopipettes with different states at +1 V. Nanochannel in state 1 and state 2 was negatively rectified. Upon the treatment of 1 μm Zn^{2+} (30 min) to induce sub-2 cleavage, the state of the nanochannel changed from negatively rectified to positively rectified (state 3 and 4), as the carboxyl tail at 5'-end of substrate 2 was cut to expose amino groups. The repulsion between amino groups and positively charged MB induced increased MB delivery into single HeLa cells. Scale bar: 20 μm. State 1: hydrophobization, negative rectification. State 2: hydrophilization, negative rectification. State 3: hydrophobization, positive rectification. State 4: hydrophilization, positive rectification. Scale bar: 20 μm.

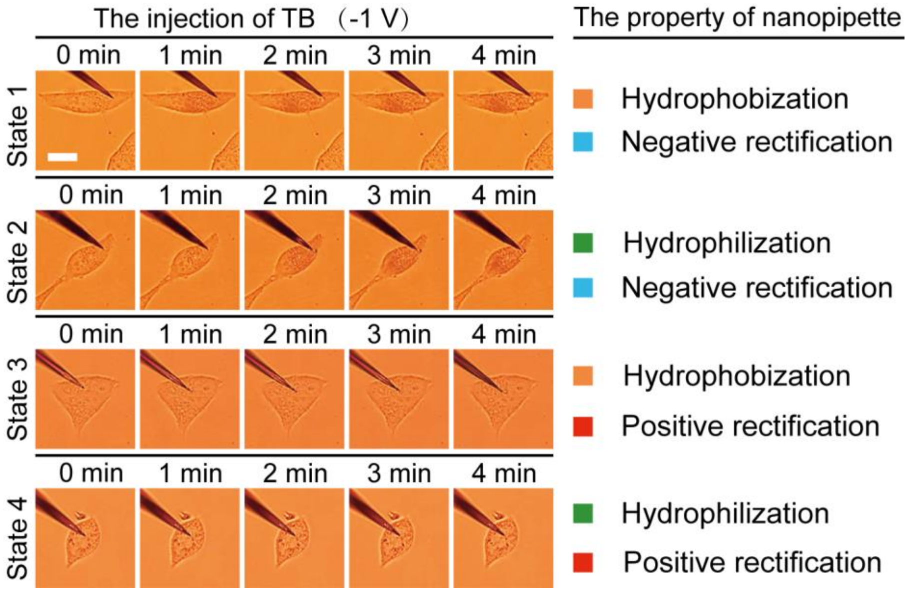

Supplementary Fig. 21 | Microscopic cell images showing the outflow of TB dye molecules into single living HeLa cells. TB (4%) solution was injected into the tip of nanopipettes with different states at -1 V. Nanochannel in state 1 and state 2 was negatively rectified, as the repulsion between the inner surface and negatively charged TB induced increased TB delivery into single HeLa cells. In contrast, positively rectified state 3 and 4 showed less delivery of TB. Scale bar: 20 μm. State 1: hydrophobization, negative rectification. State 2: hydrophilization, negative rectification. State 3: hydrophobization, positive rectification. State 4: hydrophilization, positive rectification. Scale bar: 20 μm.

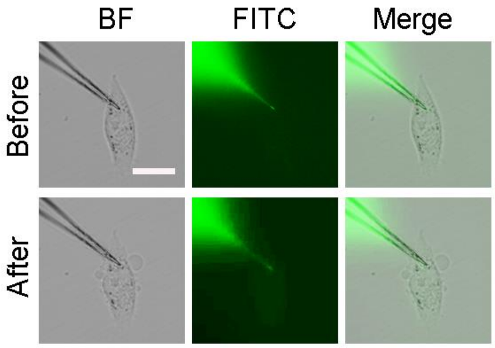

Supplementary Fig. 22 | Microscopic cell images showing the outflow of FITC dye molecules into single living HeLa cells. FITC (5 mg/mL) solution was injected into the tip of bare nanopipettes at -1 V for 20 s. Scale bar: 20 μm.

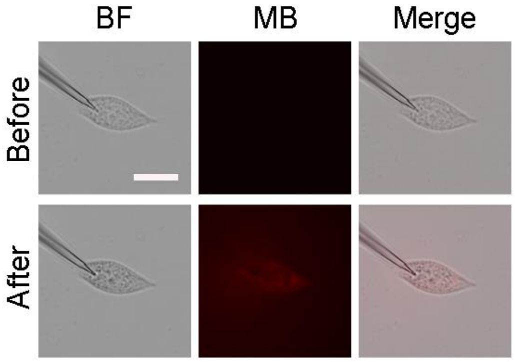

Supplementary Fig. 23 | Microscopic cell images showing the outflow of MB dye molecules into single living HeLa cells. MB (5 mg/mL) solution was injected into the tip of bare nanopipettes at +1 V for 7 min. Scale bar: 20 μm.

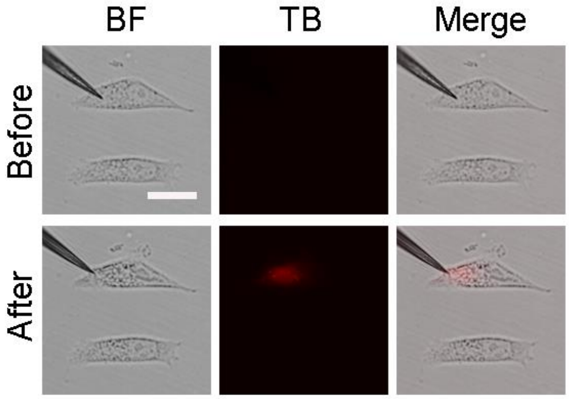

Supplementary Fig. 24 | Microscopic cell images showing the outflow of TB dye molecules into single living HeLa cells. TB (4%) solution was injected into the tip of bare nanopipettes at -1 V for 4 min. Scale bar: 20 μm.

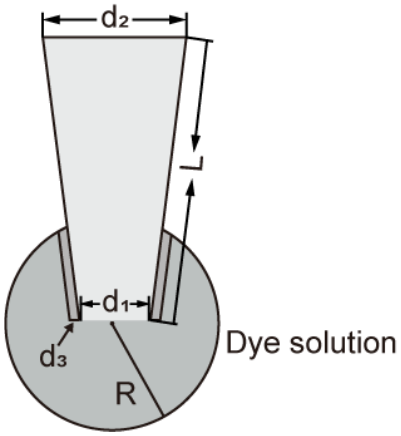

Supplementary Fig. 25 | The simulated geometry of the nanopipette. The round area at the nanopipette tip refers to the peripheral region around the tip. L, the length of the nanopipette; d_1, d_2, the diameter of the nanopipette tip and top, respectively; d_3, the thickness of the nanopipette wall. R, the diameter of the peripheral region.

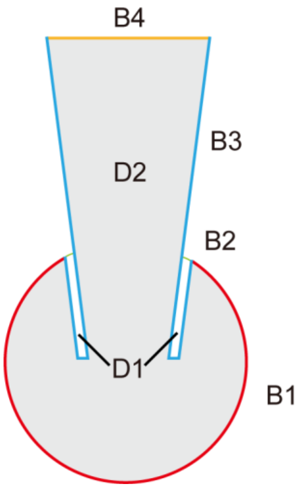

Supplementary Fig. 26 | The simulated geometry of the nanopipette (not to scale). The electric potential (V) was solved throughout the system (domains D1 and D2) using the Poisson equation (Eq. 1). The round area at the nanopipette tip refers to the dye solution around the tip, not the whole cell, and F is Faraday's constant (96485 C/mol). ε_r is the relative permittivity of the domain (ε_r = 3.75 for D1, ε_r = 78 for D2). ε_0 is the permittivity of free space, i, and c_i is the concentration of species i. The flux, j_i was solved in domain D2 only, using the Nernst-Planck equation (Eq. 2) with continuity equation (Eq. 3) where D_i refers to the diffusion coefficient, R the molar gas constant and T the temperature (298 K). The fluid velocity, u, and pressure, p, was solved for domain D2 using the Navier-Stokes equation (Eq. 4) with continuity equation (Eq. 5) where ρ refers to the solution density (1000 kg/m^3) and μ the dynamic viscosity (1.0×10^{-3} Pa·s).

$$\nabla^2 V = -\frac{F}{\varepsilon_r \varepsilon_0}\sum_i z_i c_i \tag{1}$$

$$j_i = -D_i \nabla c_i - z_i \frac{F}{RT} D_i c_i \nabla V \tag{2}$$

$$\nabla \cdot (j_i + u c_i) = 0 \tag{3}$$

$$\rho \frac{\partial u}{\partial t} + \rho (u \cdot \nabla) u = -\nabla p + \mu \nabla^2 u + F \sum_i (z_i c_i) \nabla V \tag{4}$$

$$\rho \nabla \cdot u = 0 \tag{5}$$

Initial conditions were set as the system default value. The detailed boundary conditions used in the nanopipette model were shown in Supplementary Table 5.

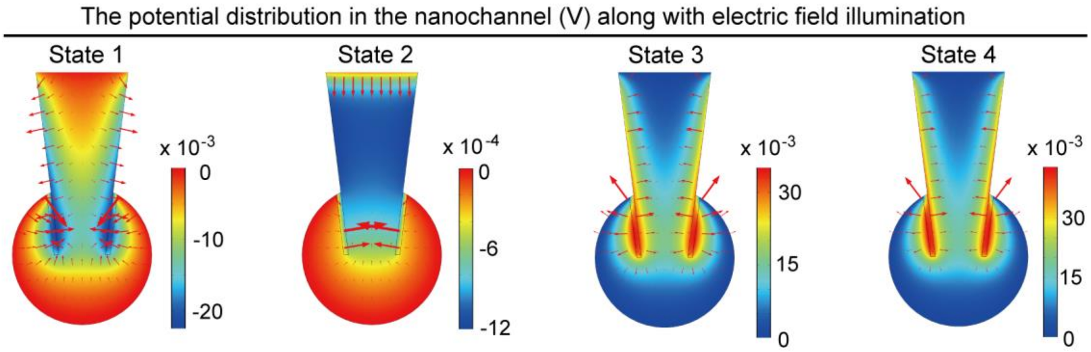

Supplementary Fig. 27 | The distribution of potential and electric field in the simulated nanopipette. When the tip of nanopipette was embedded into the plasma membrane of a single living cell to mimic the biological nanochannel, an electrostatic field in the whole model was created by the mutual coupling between the charge of TB dye molecules and the DNAzyme-functionalized nanochannel, without external potential applied at the top of the nanopipette or grounding the edge of the droplet (V0). The length of the arrow represents the magnitude of the electric field, and the direction of the arrow represents the direction of the electric field.

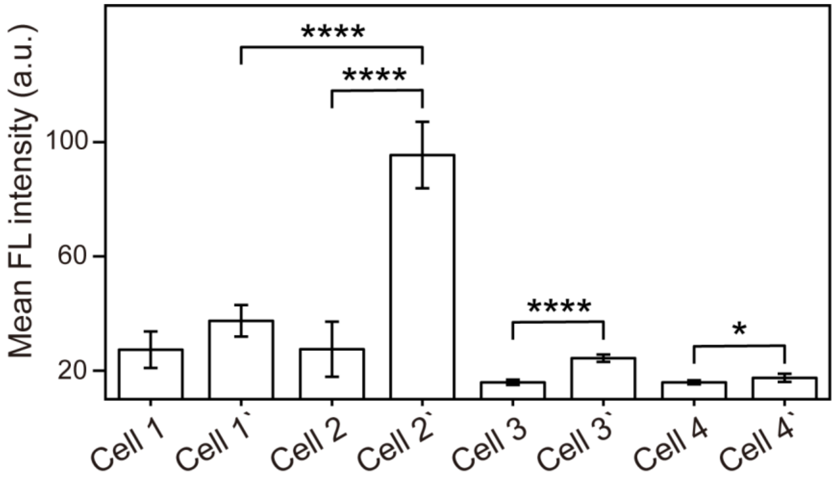

Supplementary Fig. 28 | FL intensity comparison histogram of TB before and after the treatment of TB dye molecules. The nanopipette was fixed on a holder and controlled to move along the X, Y, and Z directions to approach the single cell. Then embedded the artificial nanochannels into the plasma membrane of single living cell using the truncated tip of the nanopipettes to mimic the biological nanochannel. Cells were sent for FL imaging before and after the treatment of 2% TB dyes solution for 5 min. Significance of the mean FL intensity was assessed by t-test (n=5 independent experiments and the data were presented as the mean values ± SDs, *: $p<0.05$; **: $p<0.01$; ***: $p<0.001$; ****: $p<0.0001$). All statistics were calculated using two-tailed paired *t* test. Error bars in g represent the standard deviation of five independent experimental repeats and the measure of the centre represents their corresponding mean value. Source data are provided as a Source Data file.

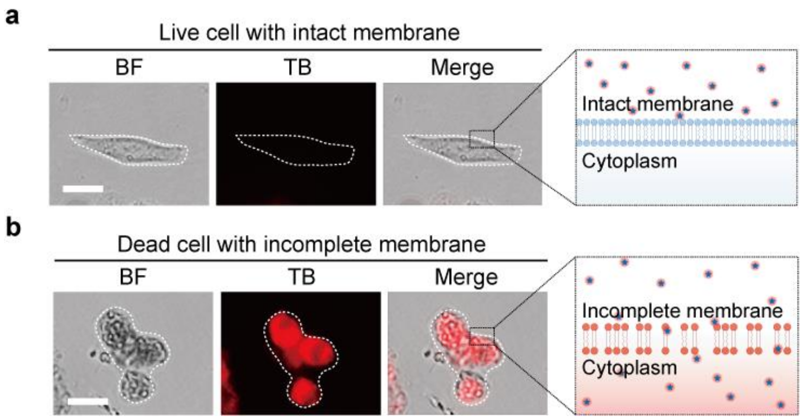

Supplementary Fig. 29 | Microscopic cell images showing TB treated cells in different states. a, Schematic illustration showing the repulsion of TB dye molecules by intact cell membrane (membrane of living cells, with no artificial nanochannel embedded into the cell membrane). **b,** Microscopic cell images showing HeLa cells before and after the treatment of TB (2%) solution for 5 min. BF: bight-field. TB: red fluorescence. Merge: mixed red & bright-field channel. Scale bar: 20 μm.

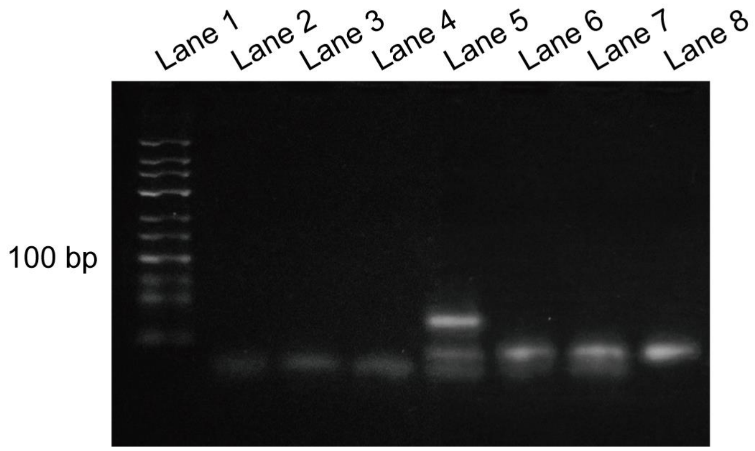

Supplementary Fig. 30 | Specific recognition between miR-21 and hp-seed evaluated by agarose gel electrophoresis. From lanes 1 to 8: ladder (lane 1), miR-21 (lane 2), mismatched miR-21 (lane 3), anti miR-21 (lane 4), mixture of hp-seed / miR-21 (lane 5), mixture of hp-seed / mismatched miR-21 (lane 6), mixture of hp-seed / anti miR-21 (lane 7), hp-seed (lane 8). The agarose gel was run in 1x Tris-Borate-EDTA buffer at 100 V for 35 min and then stained by ethidium bromide. The gel was imaged on a Bio-Rad molecular imager under blue light. The uncropped gel is shown in Source Data. Source data are provided as a Source Data file.

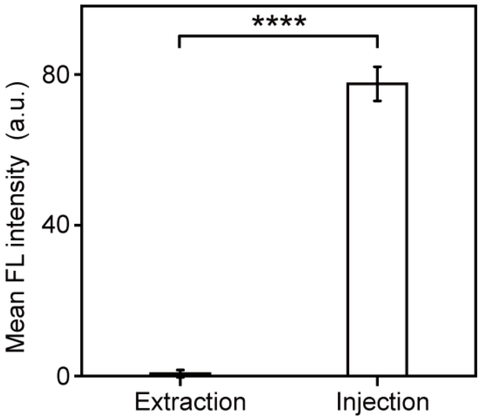

Supplementary Fig. 31 | FL intensity comparison histogram of FAM before and after miR-21 silencing. The nanopipette was embedded into a living HeLa cell. Upon application of a DC voltage (+1 V, 1 min), the miR-21 was extracted into to tip. After hybridization between miR-21 and FAM labeled antisense hairpin strand (hp-seed), the silenced miR-21 labeled by FAM was delivered into the cell via injection (-1 V, 2 min). Significance of the mean FL intensity was assessed by t-test (n=3 independent experiments and the data were presented as the mean values ± SDs, ****: $p = 0.00000863563 < 0.0001$). All statistics were calculated using two-tailed paired *t* test. Error bars in g represent the standard deviation of five independent experimental repeats and the measure of the centre represents their corresponding mean value. Source data are provided as a Source Data file.

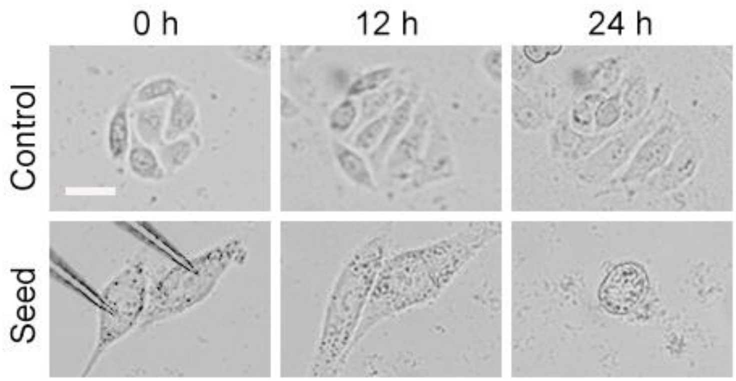

Supplementary Fig. 32 | Microscopic cell images showing the proliferation of HeLa cells treated without or with miR-21 silencing. Up: the control group without any treatment. Down: HeLa cells injected by the antisense strand against miR-21 (chain-8) solution (5 μM) under a DC voltage (-1 V, 1 min). Scale bar: 20 μm.

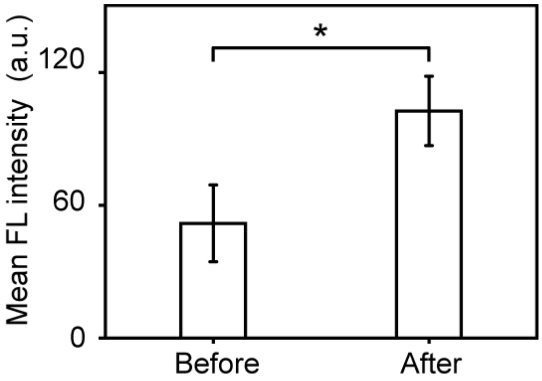

Supplementary Fig. 33 | FL intensity comparison histogram of Fluo-4 before and after the treatment of Ca^{2+}. A nanopipette tip was embedded in a living PC-12 cell. The cell was stained by Fluo-4. The fluorescence of the cell was detected before and after the treatment of Ca^{2+}. Significance of the mean FL intensity was assessed by t-test (n=3 independent experiments and the data were presented as the mean values ± SDs, *: $p = 0.02071 < 0.05$). All statistics were calculated using two-tailed paired *t* test. Error bars in g represent the standard deviation of five independent experimental repeats and the measure of the centre represents their corresponding mean value. Source data are provided as a Source Data file.

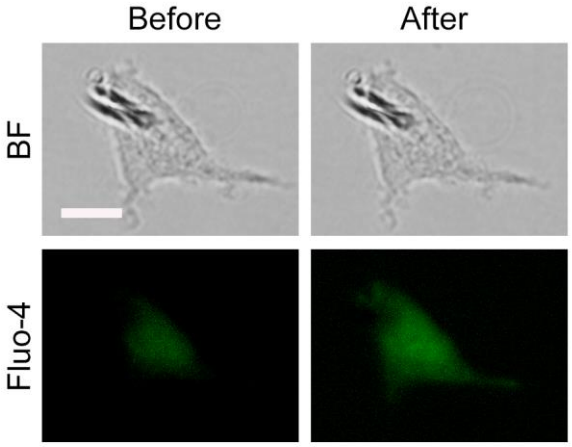

Supplementary Fig. 34 | Microscopic cell images showing the FL of fluo-4 before and after the treatment of Zn^{2+} ions. A single PC-12 cell stained by Fluo-4 and embedded by a nanopipette tip (state 1) was treated by Zn^{2+} ions, and the nanochannel transferred from negative (state 1) to positive (state 3), enabling the influx of Ca^{2+} and led to the FL enhancement of fluo-4. Scale bar: 20 μm.

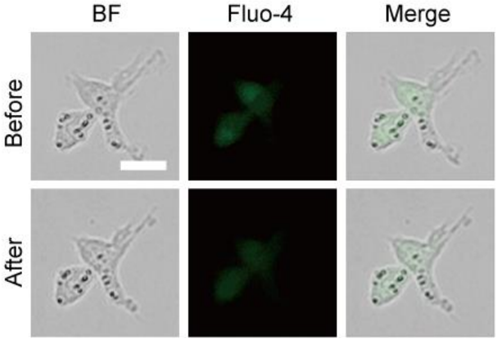

Supplementary Fig. 35 | Microscopic cell images showing the detection of Ca^{2+} based on Fluo-4 probe in PC-12 cells. Before the incubation of Fluo-4 probe, PC-12 cells were washed with PBS buffer for three times to remove serum, and then the cells were incubated with Fluo-4 probe (2 μM) for 30 min at room temperature. After that, cells were incubated for another 20 min at room temperature after washing three times with PBS buffer. Before: before the treatment of Ca^{2+}. After: after the treatment of Ca^{2+} (1 mM, 20 min). BF: bight-field. Fluo-4: green fluorescence. Merge: mixed green & bright-field channel. Scale bar: 20 μm.

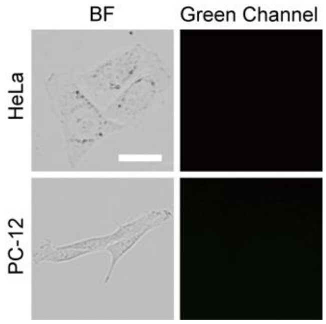

Supplementary Fig. 36 | Microscopic cell images showing the spontaneous FL of HeLa cells and PC-12 cells. HeLa cells and PC-12 cells without any treatment were sent for FL imaging under 488 nm laser light. Scale bar: 20 μm.

Supplementary Tables:

Supplementary Table 1 | State conversion of artificial nanochannels.

Order	Initial state	Final state	Reagents
**A**	Hydrophobic<br>Neg. Rec.	Hydrophilic<br>Neg. Rec.	Mg^{2+}
**B**	Hydrophilic<br>Neg. Rec.	Hydrophobic<br>Neg. Rec.	Substrate 1
**C**	Hydrophobic<br>Neg. Rec.	Hydrophobic<br>Pos. Rec.	Zn^{2+}
**D**	Hydrophobic<br>Pos. Rec.	Hydrophobic<br>Negative	Substrate 2
**E**	Hydrophobic<br>Pos. Rec.	Hydrophilic<br>Pos. Rec.	Mg^{2+}
**F**	Hydrophilic<br>Pos. Rec.	Hydrophobic<br>Pos. Rec.	Substrate 1
**G**	Hydrophilic<br>Neg. Rec.	Hydrophilic<br>Pos. Rec.	Zn^{2+}
**H**	Hydrophilic<br>Pos. Rec.	Hydrophilic<br>Neg. Rec.	Substrate 2
**I**	Hydrophobic<br>Negative	Hydrophilic<br>Pos. Rec.	Mg^{2+}<br>Zn^{2+}
**J**	Hydrophilic<br>Pos. Rec.	Hydrophobic<br>Neg. Rec.	Substrate 1<br>Substrate 2
**K**	Hydrophilic<br>Neg. Rec.	Hydrophobic<br>Pos. Rec.	Substrate 1<br>Zn^{2+}
**L**	Hydrophobic<br>Pos. Rec.	Hydrophilic<br>Neg. Rec.	Mg^{2+}<br>Substrate 2

*Positive rectification (Pos. Rec). Negative rectification (Neg. Rec.).

Supplementary Table 2 | Oligonucleotides sequences.

Name	Sequence (5`------------3`)	Tm (°C)
DNAzyme 1	NH_2-/iSp 18/CAGTACCTTCAGCAACATCGATCGGAAGCCAGTTG	66.54
Substrate 1	Cholesteryl-CAACUGGCUrUrGGAAGGUACUG	56.04
DNAzyme 2	NH_2-/iSp 18/ATAGTTTCTCCGAGCCGGTCGAAACTTCTCTACCTGCAA-NH_2	67.78
Substrate 2	COOH-TTGCAGGTAGAGAAGT/rA/GGAAACTAT	54.48
Hp-seed	FAM-CCGTTCTATCAACATCAGTCTGATAAGCTATAGAACGG-BHQ	61.41
MicroRNA-21 (miR-21)	UAGCUUAUCAGACUGAUGUUGA	60.00
Mismatched miR-21	UAGCUUAUCAGACAGAUGUUCA	60.00
Anti miR-21	UCAACAUCAGUCUGAUAAGCUA	60.00
Chain-1	FAM-CAGTACCTTCAGCAACATCGATCGGAAGCCAGTTG-SH	66.54
Chain-2	NH_2-CAGTACCTTCAGCAACATCGATCGGAAGCCAGTTG	66.54
Chain-3	NH_2-/iSp 18/CAGTACCTTCAGCAACATCGATCGGAAGCCAGTTG	66.54
Chain-4	NH_2-/iSp 18/ATAGTTTCTCCGAGCCGGTCGAAACTTCTCTACCTGCAA-NH_2	67.78
Chain-5	NH_2-ATAGTTTCTCCGAGCCGGTCGAAACTTCTCTACCTGCAA-NH_2	67.78
Chain-6	NH_2-/iSp 18/CTGAACTTCCAGCAACATCGATCGGAACGCATTGG	67.05
Chain-7	NH_2-/iSp 18/AATGTAACTCCGAGCCGGTCGAACATTCTATCCCTCGAA-NH_2	67.69
Chain-8	CCGTTCTATCAACATCAGTCTGATAAGCTATAGAACGG	61.41

*DNAzyme 1 and substrate 1 are Mg^{2+}-specific used to build a DNA double-chain control switch for inner surface wettability regulation. DNAzyme 2 and substrate 2 are Zn^{2+}-specific used to build a DNA double-chain control switch for inner surface charge regulation. Hp-seed is used for detecting and silencing microRNA-21 (miR-21) in single HeLa cells, and the FL of FAM is quenched by the adjacent BHQ group via the hairpin structure of the antisense strand. The addition of miR-21 can open the loop part of hp-seed and release the green FL of FAM. Chain-1 is used for functionalization of nanopipette via Au cladding modification (Supplementary Fig. 4). Chain-2 and chain-3 are used for inner surface modification of nanopipette via the interaction between aldehyde and amino group with different length (Supplementary Fig. 5, 6). Chain-4 and chain-5 are used to test the influence of DNAzyme length on the charge of nanopipette (Supplementary Fig. 7). Chain-6 and chain-7 are inactive DNAzymes used to test the availability and specificity of the DNAzyme-based switching (Supplementary Fig. 10, 14). Chian-8 is used for silencing microRNA-21 in HeLa cells (Supplementary Fig. 32).

*iSp 18: Spacer 18. rUrG: adenine ribonucleotide. rA: adenine ribonucleotide.

Supplementary Table 3 | Parameters for the fabrication of nanopipettes using a P-2000 laser.

	Heat	Fil	Vel	Del	Pul
Line 1	680	3	22	135	95

Supplementary Table 4 | The detailed simulation parameters of the nanopipette model.

Name	Expression	Value	Description
d_1	100[nm]	1E-7 m	Diameter 1
d_2	200[nm]	2E-7 m	Diameter 2
d_3	10[nm]	1E-8 m	Diameter 3
**L**	400[nm]	4E-7 m	Length
**R**	150[nm]	1.5E-7 m	Radius
**V**	0[mV]	0 V	Potential

Supplementary Table 5 | The detailed boundary conditions used in the nanopipette model.

Boundary	Poisson	Nernst-Planck	Navier-Stokes
**B1**	$V = 0\ \mathrm{V}$		$(-\nabla p + \mu \nabla^2 u) \cdot \vec{\mathbf{n}} = 0$
**B2**	$\vec{\mathbf{n}} \cdot \left({E_2}/{\varepsilon_2} \right) = 0$		
**B3**	$\sigma = \vec{\mathbf{n}} \cdot \left({E_1}/{\varepsilon_1} - {E_2}/{\varepsilon_2} \right)$	$-\vec{\mathbf{n}} \cdot j_i = 0$	$u = 0$
**B4**	$V = 0\ \mathrm{V}$		$(-\nabla p + \mu \nabla^2 u) \cdot \vec{\mathbf{n}} = 0$
