# Supplementary material for: Rectifying artificial nanochannels with multiple interconvertible permeability states
Source: Nat Commun. 2024 Mar 6;15:2051. doi: 10.1038/s41467-024-46312-w (PMC10918189; doi:10.1038/s41467-024-46312-w)
Supplement: Supplementary file 3 — Reporting Summary [file 41467_2024_46312_MOESM3_ESM.pdf]

nature portfolio

Corresponding author(s): Ruo-Can Qian, Yi Lu

Last updated by author(s): Jan 22, 2024

Reporting Summary

Nature Portfolio wishes to improve the reproducibility of the work that we publish. This form provides structure for consistency and transparency in reporting. For further information on Nature Portfolio policies, see our Editorial Policies and the Editorial Policy Checklist.

Statistics

For all statistical analyses, confirm that the following items are present in the figure legend, table legend, main text, or Methods section.

n/a	Confirmed	
☐	☒	The exact sample size (n) for each experimental group/condition, given as a discrete number and unit of measurement
☐	☒	A statement on whether measurements were taken from distinct samples or whether the same sample was measured repeatedly
☐	☒	The statistical test(s) used AND whether they are one- or two-sided<br>*Only common tests should be described solely by name; describe more complex techniques in the Methods section.*
☐	☒	A description of all covariates tested
☐	☒	A description of any assumptions or corrections, such as tests of normality and adjustment for multiple comparisons
☐	☒	A full description of the statistical parameters including central tendency (e.g. means) or other basic estimates (e.g. regression coefficient) AND variation (e.g. standard deviation) or associated estimates of uncertainty (e.g. confidence intervals)
☐	☒	For null hypothesis testing, the test statistic (e.g. F, t, r) with confidence intervals, effect sizes, degrees of freedom and P value noted<br>*Give P values as exact values whenever suitable.*
☒	☐	For Bayesian analysis, information on the choice of priors and Markov chain Monte Carlo settings
☒	☐	For hierarchical and complex designs, identification of the appropriate level for tests and full reporting of outcomes
☒	☐	Estimates of effect sizes (e.g. Cohen's d, Pearson's r), indicating how they were calculated

Our web collection on statistics for biologists contains articles on many of the points above.

Software and code

Policy information about availability of computer code

Data collection	The nanopipette was fixed under the microscope for electrochemical measurements by a holder (Axon Instruments, Union City, CA). All ionic current recordings were carried out using an Axopatch 200B low-noise amplifier and an Axon Digidata 1550A low-noise data acquisition system (Molecular Devices, Sunnyvale, CA). Microscopic cell images were collected using IS-Elements BR 5.30.05 64. The Supplementary Movies were collected using oCam. All the FL images were obtained using a water dipping objective (60×) on a laser scanning confocal microscope (Nikon A1R., Japan). All software used in this study for data collection are either commercially available or open source.
Data analysis	The ionic current data were carried out using Clampfit 10.6 and then plotted by Origin (2021) and further imported to Adobe Illustrator (2021). Numerical simulations of the electro-osmotic flow inside the nanopipette were performed using COMSOL Multiphysics 5.4 (COMSOL AB, Stockholm, Sweden) to show the detailed profiles of the voltage and concentration distribution of TB dyes. The 2D axisymmetric model was designed for mimicking the cone-shaped nanopipettes based on the electrostatics module and the creeping flow module, can be found in the Supplementary Information. All reported errors in the manuscript and supplementary information represent one standard deviation. Statistical analyses were carried out using Excel (Microsoft Office 365) and Origin (2021). The figures were plotted by Origin (2021) and further imported to Adobe Illustrator (2021).

For manuscripts utilizing custom algorithms or software that are central to the research but not yet described in published literature, software must be made available to editors and reviewers. We strongly encourage code deposition in a community repository (e.g. GitHub). See the Nature Portfolio guidelines for submitting code & software for further information.

Data

Policy information about availability of data

All manuscripts must include a data availability statement. This statement should provide the following information, where applicable:

- Accession codes, unique identifiers, or web links for publicly available datasets
- A description of any restrictions on data availability
- For clinical datasets or third party data, please ensure that the statement adheres to our policy

The main data supporting this study's results are avaiable within the paper and its Supplementary Information. Source data are provided with this paper. The raw data has been provided in source data and has been deposited in Figshare database at https://figshare.com/s/7f0e66ea96f7ac4f557a. Additional relevant information is available from the corresponding author.

Research involving human participants, their data, or biological material

Policy information about studies with human participants or human data. See also policy information about sex, gender (identity/presentation), and sexual orientation and race, ethnicity and racism.

Reporting on sex and gender	n/a
Reporting on race, ethnicity, or other socially relevant groupings	n/a
Population characteristics	n/a
Recruitment	n/a
Ethics oversight	n/a

Note that full information on the approval of the study protocol must also be provided in the manuscript.

Field-specific reporting

Please select the one below that is the best fit for your research. If you are not sure, read the appropriate sections before making your selection.

☒ Life sciences ☐ Behavioural & social sciences ☐ Ecological, evolutionary & environmental sciences

For a reference copy of the document with all sections, see nature.com/documents/nr-reporting-summary-flat.pdf

Life sciences study design

All studies must disclose on these points even when the disclosure is negative.

Sample size	The sample analysis, at least 3 samples were tested for each state (state 1, state 2, state 3 and state 4). No statistical methods were used to determine sample size. The sample size were decided according to a previous study using the same cohorts of samples. The selection of the sample size took into account both statistical significance and the timeliness of validating experimental performance in exploratory experiments.
Data exclusions	No data were excluded from the analysis.
Replication	Experiments were repeated three independently times. All attempts of replication were successful.
Randomization	Cells were randomly allocated into control and experimental groups for cell injection.
Blinding	Data collection and analysis were not blinded. Blinding was not relevant because the study is a pilot investigation. The main purpose of the study was to test the feasibility of DNAzyme-functionalized artificial biomimetic nanochannels to realize precise control of the inner surface wettability and charge between four different states under external stimuli. Blinding might complicate the experimental setup or workflow, potentially leading to inefficiencies in this pilot experiment.

Reporting for specific materials, systems and methods

We require information from authors about some types of materials, experimental systems and methods used in many studies. Here, indicate whether each material, system or method listed is relevant to your study. If you are not sure if a list item applies to your research, read the appropriate section before selecting a response.

Materials & experimental systems

n/a	Involved in the study	
☒	☐	Antibodies
☐	☒	Eukaryotic cell lines
☒	☐	Palaeontology and archaeology
☒	☐	Animals and other organisms
☒	☐	Clinical data
☒	☐	Dual use research of concern
☒	☐	Plants

Methods

n/a	Involved in the study	
☒	☐	ChIP-seq
☒	☐	Flow cytometry
☒	☐	MRI-based neuroimaging

Eukaryotic cell lines

Policy information about cell lines and Sex and Gender in Research

Cell line source(s)	HeLa cervical cancer cells and rat pheochromocytoma 12 (PC-12) cells were purchased from bluefbio (Shanghai) Biology Technology Development Co., Ltd. (Shanghai, China).
Authentication	No authentication has been used.
Mycoplasma contamination	There was no contamination during experiments.
Commonly misidentified lines (See ICLAC register)	No commonly misidentified lines were used in this study
