# Supplementary material for: Rectifying artificial nanochannels with multiple interconvertible permeability states
Source: Nat Commun. 2024 Mar 6;15:2051. doi: 10.1038/s41467-024-46312-w (PMC10918189; doi:10.1038/s41467-024-46312-w)
Supplement: Supplementary file 4 — Description of Additional Supplementary Files [file 41467_2024_46312_MOESM4_ESM.pdf]

**Description of Additional Supplementary Files**

**File Name: Supplementary Movie 1**
**Description:** The insertion of a nanopipette into a single living HeLa cell.

**File Name: Supplementary Movie 2**
**Description:** The outflow of FITC dyes into a single HeLa cell at -1 V in a state 1 nanochannel.

**File Name: Supplementary Movie 3**
**Description:** The outflow of FITC dyes into a single HeLa cell at -1 V in a state 2 nanochannel.

**File Name: Supplementary Movie 4**
**Description:** The outflow of FITC dyes into a single HeLa cell at -1 V in a state 3 nanochannel.

**File Name: Supplementary Movie 5**
**Description:** The outflow of FITC dyes into a single HeLa cell at -1 V in a state 4 nanochannel.

**File Name: Supplementary Movie 6**
**Description:** The outflow of MB dyes into a single HeLa cell at 1V in a state 1 nanochannel.

**File Name: Supplementary Movie 7**
**Description:** The outflow of MB dyes into a single HeLa cell at 1 V in a state 2 nanochannel.

**File Name: Supplementary Movie 8**
**Description:** The outflow of MB dyes into a single HeLa cell at 1 V in a state 3 nanochannel.

**File Name: Supplementary Movie 9**
**Description:** The outflow of MB dyes into a single HeLa cell at 1 V in a state 4 nanochannel.

**File Name: Supplementary Movie 10**
**Description:** The outflow of TB dyes into a single HeLa cell at -1 V in a state 1 nanochannel.

**File Name: Supplementary Movie 11**
**Description:** The outflow of TB dyes into a single HeLa cell at -1 V in a state 2 nanochannel.

**File Name: Supplementary Movie 12**
**Description:** The outflow of TB dyes into a single HeLa cell at -1 V in a state 3 nanochannel.

**File Name: Supplementary Movie 13**
**Description:** The outflow of TB dyes into a single HeLa cell at -1 V in a state 4 nanochannel.
