# Supplementary figures and images for: Rectifying artificial nanochannels with multiple interconvertible permeability states
Source: Nat Commun. 2024 Mar 6;15:2051. doi: 10.1038/s41467-024-46312-w (PMC10918189; doi:10.1038/s41467-024-46312-w)

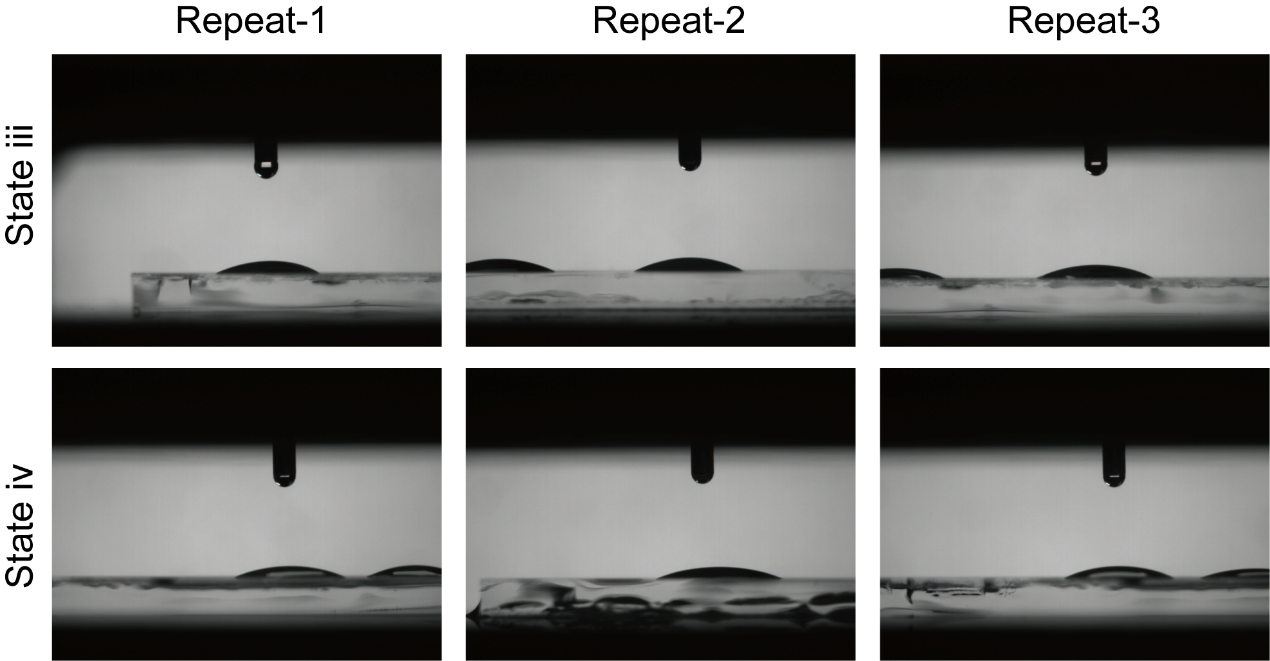

Supplement: Supplementary file 18 — Source Data [file 41467_2024_46312_MOESM18_ESM.zip › SI Fig. 12 contact angle.tif]

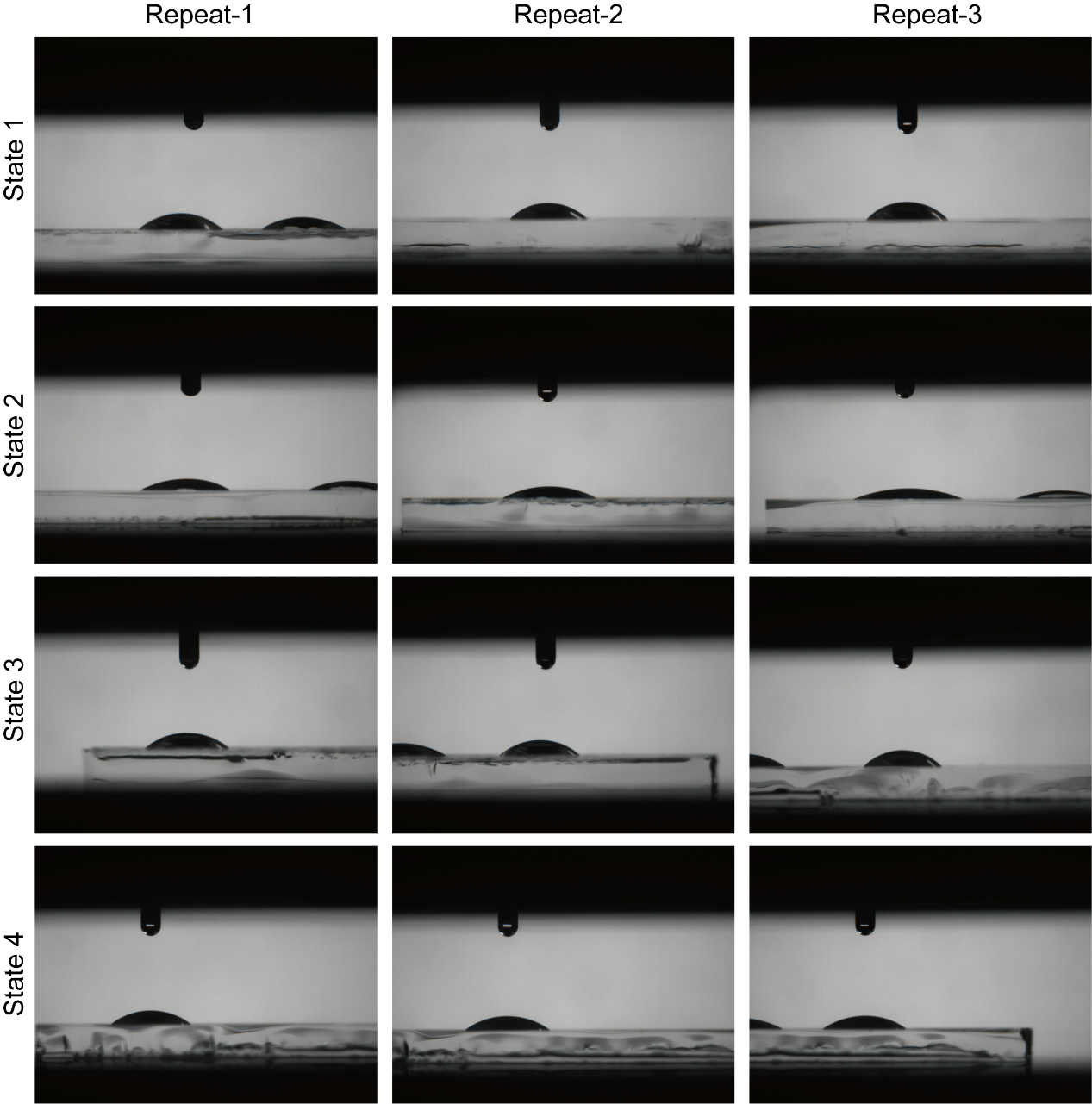

Supplement: Supplementary file 18 — Source Data [file 41467_2024_46312_MOESM18_ESM.zip › SI Fig. 16 contact angle.tif]

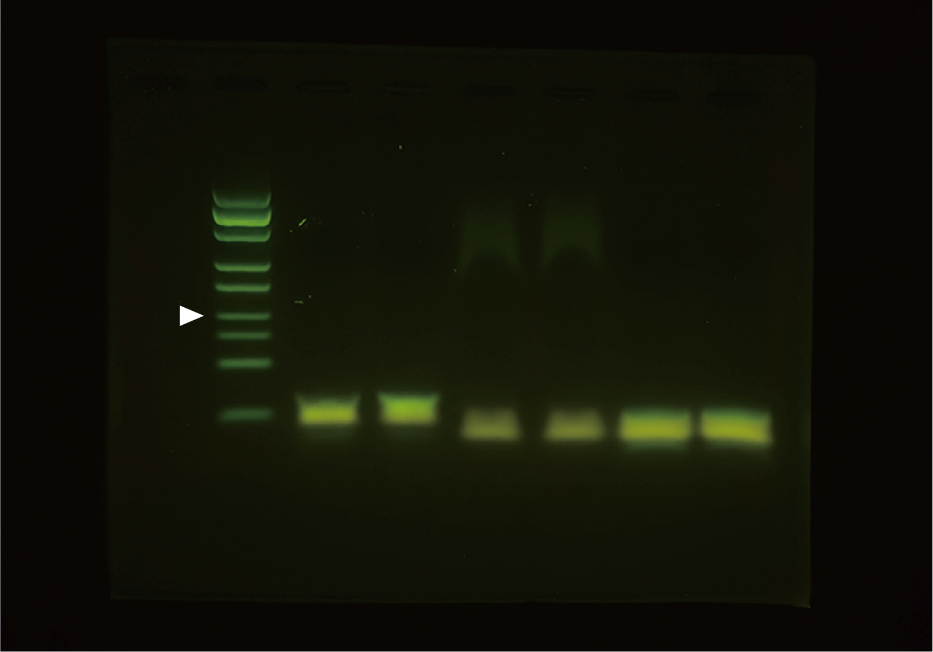

Supplement: Supplementary file 18 — Source Data [file 41467_2024_46312_MOESM18_ESM.zip › SI Fig. 17 gel.tif]

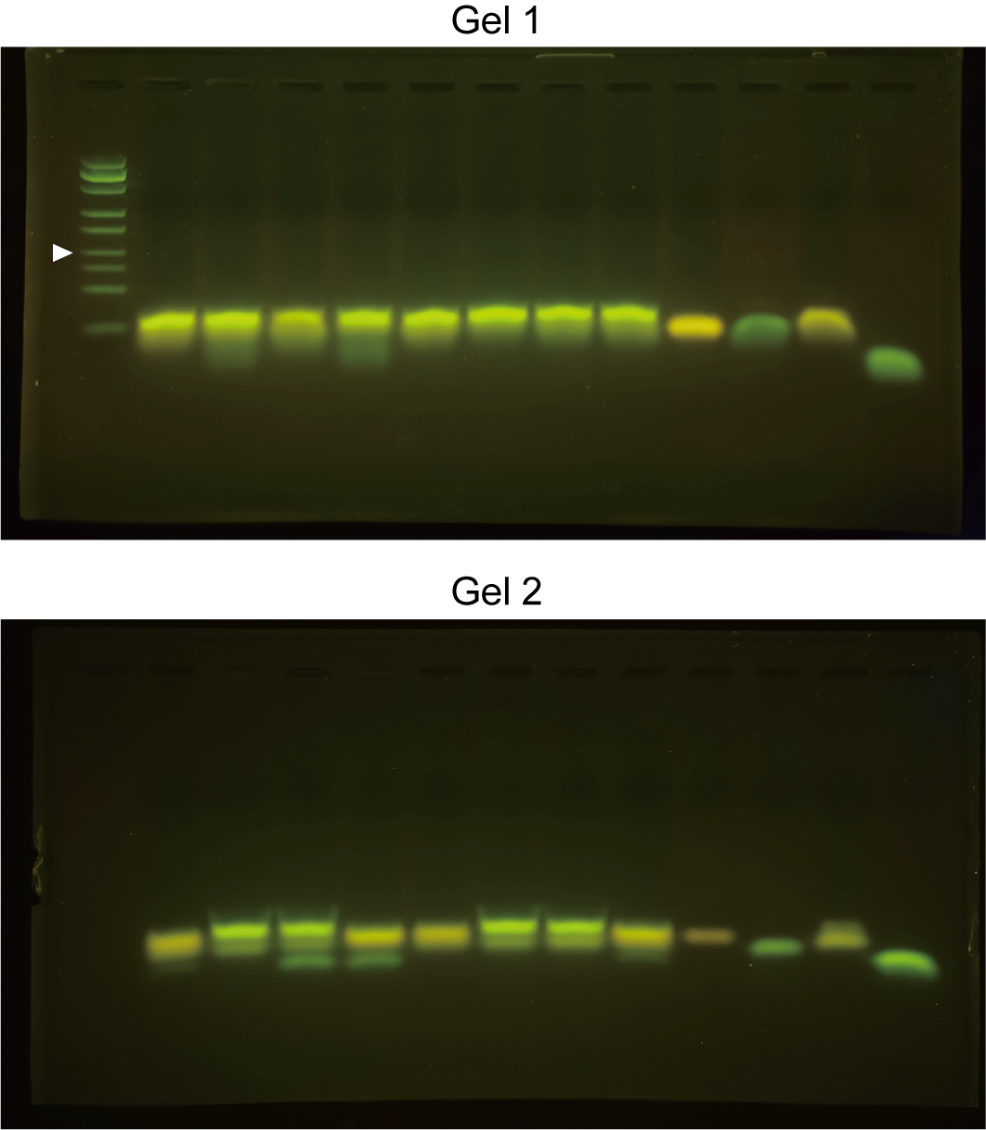

Supplement: Supplementary file 18 — Source Data [file 41467_2024_46312_MOESM18_ESM.zip › SI Fig. 18 gel.tif]

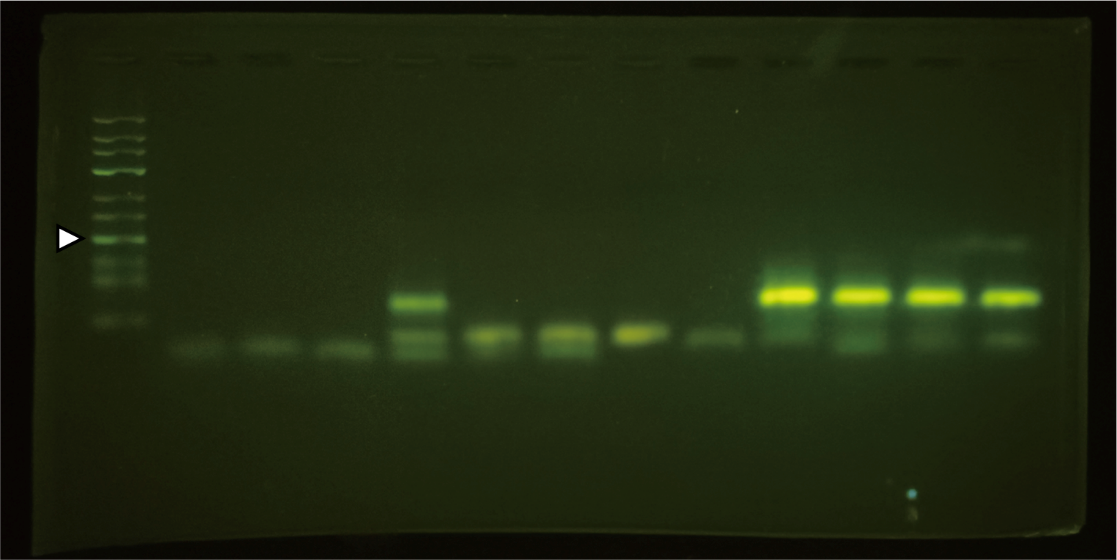

Supplement: Supplementary file 18 — Source Data [file 41467_2024_46312_MOESM18_ESM.zip › SI Fig. 30 gel.tif]

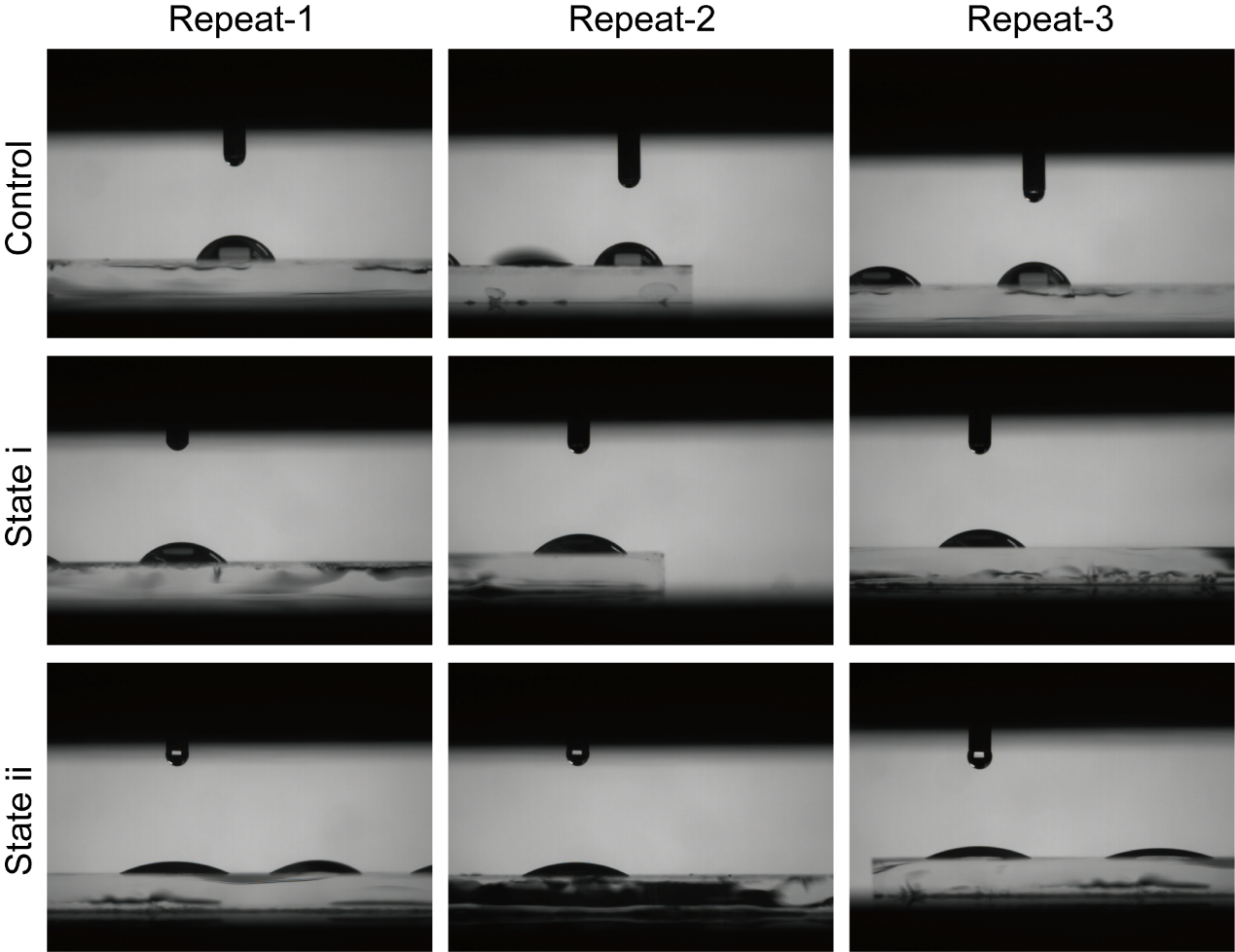

Supplement: Supplementary file 18 — Source Data [file 41467_2024_46312_MOESM18_ESM.zip › SI Fig. 8 contact angle.tif]

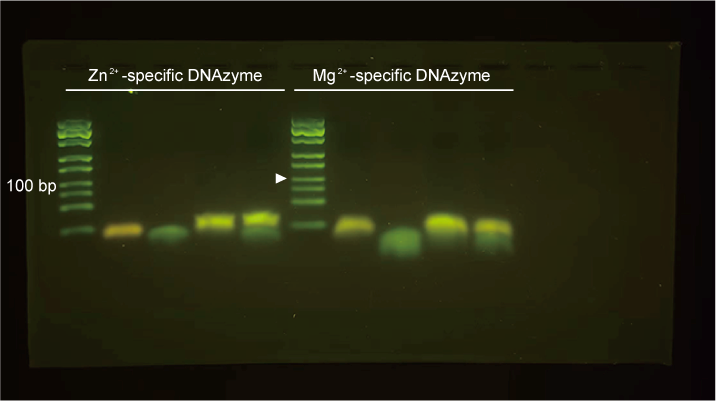

Supplement: Supplementary file 18 — Source Data [file 41467_2024_46312_MOESM18_ESM.zip › SI Fig. 9 & 13 gel.tif]
